# Robotic-Assisted Fixation and Cementation for Sacral Insufficiency Fractures: A Case Series and Technical Note

**DOI:** 10.3390/jcm15135104

**Published:** 2026-06-30

**Authors:** Gal Barkay, Maria Auron, Ohad Einav, Ahmad Shahwan, Josh E. Schroeder

**Affiliations:** 1 Department of Orthopedic Surgery, Hadassah Medical Center, Jerusalem 9371125, Israel; 2 School of Medicine, Hebrew University, Jerusalem 9190401, Israel

**Keywords:** sacral fracture, robotics, osteoporosis

## Abstract

**Background:** The prevalence of sacral insufficiency fractures resulting from minor trauma has been on the rise in parallel with the globally aging population. For similar injuries typical to the elderly population, such as hip fractures, surgery and early mobilization have been shown to improve postoperative mortality and morbidity rates. As such, there has been a recent increase in the literature in studies advocating for early surgical fixation for sacral insufficiency fractures. However, traditional fluoroscopic techniques are technically demanding and bear an inherent complication risk even in experienced hands. Robotic-assisted surgery has emerged as a promising technological advancement in spinal and pelvic surgery. We share our experience with this surgical technique. **Methods:** We conducted a retrospective analysis of five consecutive patients with sacral insufficiency fractures who failed non-operative management. Using the Mazor X robotic system, patients underwent CT-planned, guided placement of fenestrated sacroiliac screws followed by cement augmentation. Primary outcomes included surgical time, radiation exposure, complications, and mobilization, with a minimum three-month follow-up. **Results:** The cohort consisted of five females with a mean age of 78 years. The mean operative time was 36 min (15–47), and the median fluoroscopy count was 13 shots (6–19). All patients reported significant pain relief and achieved successful mobilization on postoperative day 1. No operative or postoperative complications were recorded. **Conclusions:** This pilot study suggests that robotic-assisted percutaneous sacroiliac fixation with cement augmentation is a safe, efficient, and minimally invasive approach for the treatment of sacral insufficiency fractures. The precision of the robotic system facilitates stable fixation, providing immediate pain relief and early mobilization with a favorable complication profile. Further studies should be performed to verify these findings.

## 1. Background

With the aging of the population, the prevalence of sacral insufficiency fractures occurring from minor trauma has been on the rise [1,2]. Patients with injuries characteristically present with acute onset, such as debilitating low back pain, often experience a profound decline in functional independence. Traditionally, these injuries have been treated with bed rest, pain control and gradual mobilization. However, prolonged immobilization in the elderly is highly detrimental and can be associated with severe systemic complications that can significantly increase both morbidity and mortality. For similar injuries typical to the elderly population such as hip fractures, surgery and early mobilization have been shown to improve postoperative mortality and morbidity rates as well as pain scores [3,4,5]. As such, there has been a recent increase in the literature advocating for early surgical fixation for sacral insufficiency fractures in this patient population [6,7].

Traditional freehand techniques, relying on fluoroscopic guidance, are technically demanding and bear an inherent complication risk even in the most experienced hands. Robotic-assisted surgery has emerged as a promising technological advancement in spinal and pelvic surgery, potentially offering advantages such as enhanced accuracy of implant placement as well as decreased radiation exposure and surgical time [8,9,10]. This technology utilizes stereotactic guidance based on preoperative 3d imaging, thus overcoming possible errors in freehand fixation techniques.

The clinical challenge of sacral fixation is further exacerbated by the poor bone mineral density inherent in this patient demographic. In severely osteoporotic bone, conventional screw fixation alone may be prone to failure due to insufficient pull-out strength, particularly when subjected to the complex multi-axial forces of the pelvic ring. The integration of cement augmentation addresses this limitation by possibly providing immediate internal stability and reinforcing the bone–screw interface. This hybrid approach seeks to combine the surgical precision of robotic navigation with the mechanical durability of polymethylmethacrylate (PMMA), theoretically allowing for the immediate weight-bearing necessary to avoid the systemic complications of prolonged immobility.

However, the clinical implementation of cement augmentation introduces unique technical challenges, most notably the risk of cement extravasation into adjacent neurovascular structures. The sacrum features highly complex and variable osseous anatomy, with narrow corridors situated in immediate proximity to the neural structures and major iliac vessels. A minor cortical breach or trajectory deviation during screw insertion can create a path of least resistance, potentially leading to severe cement leakage into the neural foramina or pelvic cavity. Robotic-assisted surgery directly addresses this safety concern by providing ultra-precise, image-guided trajectory planning and real-time execution [11]. By ensuring that the fenestrated screw remains entirely within the safe intraosseous corridor, robotic navigation establishes a secure path for targeted cement delivery, minimizing the likelihood of structural breach and subsequent leakage.

Furthermore, while the independent clinical benefits of both robotic navigation and cement-augmented fixation have been separately documented, there remains a distinct scarcity of studies in the literature evaluating their concurrent use in a singular clinical workflow. Biomechanical studies have established that cement augmentation significantly enhances screw anchorage and limits construct displacement within the osteoporotic posterior pelvic [12]. Yet, the vast majority of the current clinical literature focuses on either evaluating the accuracy of robotic pelvic screw placement alone or measuring the mechanical outcomes of manually placed cemented screws under conventional fluoroscopy. There is an evident gap in comprehensive clinical evidence outlining a unified, reproducible, and standardized surgical protocol that seamlessly integrates robotic precision with controlled PMMA augmentation.

We present our experience with a robotic-assisted sacral fixation technique with cement augmentation in a series of five patients and provide technical insight on the surgical technique, specifically detailing the workflow required for optimal safety and efficacy.

## 2. Methods

Approval from the institutional review board no. HMO-0355-25 was obtained prior to data collection and analysis. Waiver of the requirement for consent was granted due to the nature of the study. This is a retrospective analysis of prospectively collected data from a cohort of patients using the computerized database of Hadassah Medical Center, Jerusalem, Israel. For patients with incomplete data, access to the nationwide medical record platform was obtained and data was extracted. Patients included were those who underwent robotic-assisted sacroiliac fixation with cement augmentation for a sacral insufficiency fracture. All patients failed a trial of non-operative management and were significantly debilitated by their injury. A trial of non-operative treatment included a strict regimen of multimodal pain management medications (including acetaminophen, non-steroidal anti-inflammatory drugs, and targeted opioid therapy). This was coupled with attempted mobilization with our physiotherapy team, with meticulous continuous assessment of the patients’ functional status and VAS pain scores. Failure of non-operative treatment was defined as the patient’s failure to progressively and functionally improve over a minimum period of 7 days. Excluded were patients with a pathology that was non-traumatic, such as a tumor or infection, as well as patients with incomplete medical records. Demographic data collected included age, gender and patient comorbidities. Hospitalization data included neurological status, additional injuries, patient complications, length of stay and physio reports. Surgical data included time of surgery and radiation exposure. Patients’ fracture pattern on presentation was assessed using computed tomography (CT). Fracture type was classified as a complete unilateral or bilateral sacral fracture. Due to the nature of our service, radiology reports for imaging studies were not readily available and hence were not included in the study. All imaging studies were independently reviewed, classified, and adjudicated by the attending senior orthopedic spine surgical team to determine fracture patterns and laterality. Intraoperative screw placement was assessed intraoperatively using standard multi-planar fluoroscopy consisting of anterior–posterior(AP)+Inlet+Outlet X-rays of the pelvis. All patients were given complete weight-bearing privileges and were mobilized on postoperative day 1 by our physiotherapy team, focusing on transferring from bed to chair, and progressing to walker-assisted ambulation as tolerated. Radiographic assessment on follow-up was done with upright radiographs at 2 weeks, 6 weeks and 3 months. Radiographic parameters assessed on follow-up were maintenance of pelvic alignment as well as hardware integrity. Minimum patient clinical and radiographic follow-up for radiographic analysis was 3 months. Outcome measures were superficial or deep surgical wound infection, or mechanical wound dehiscence requiring secondary surgical debridement or intervention, hardware failure defined as evidence of screw breakage, peri-implant loosening, or structural failure of the cement–bone interface and radiographic or clinical evidence of fracture non-union, defined as persistent fracture line lucency combined with persistent mechanical pain at the 3-month mark or later. Outpatient clinic follow-up X-rays were performed at 6 weeks and 3 months. Further clinic follow-up and advanced CT imaging were performed at the treatment team’s discretion following discussion with the patient.

## 3. Surgical Technique

Preoperatively, a thin-slice (1 mm) CT scan is performed for screw planning and CT to flouro intraoperative registration. Bilateral 8.5 mm sacroiliac screws (Solera, Medtronic, Minneapolis, MN, USA) are planned perpendicular to the fracture line with the length of the screw reaching the S1 body to allow for optimal fixation. Care is taken at this point to ensure that there is a safe pathway for cement injection with no potential for cement leakage to the neuroforamina or the pelvis. Following anesthesia, the patient is positioned prone on a radiolucent table. Pressure points are padded for patient safety. The patient is draped and a PSIS pin is inserted to mount the robotic navigation system (Mazor X stealth addition, Medtronic, Minneapolis, MN, USA). AP and oblique flouro shots are obtained for registration and subsequent CT to flouro registration is performed. Registration is verified by the lead surgeon. Screw trajectories are then drilled and tapped under navigation guidance. Navigation-guided drilling is performed with a 3 mm navigated drill followed by a 6.5 mm navigation-guided tap, ensuring exact adherence to the pre-planned trajectory. Fenestrated screws are inserted on k-wires under flouro guidance with cement towers attached. Screw placement is assessed with AP, Inlet and outlet flouro views. Crucially, the PMMA cement is mixed and allowed to reach a highly viscous, dough-like consistency prior to injection to mitigate the risk of extravasation. Cement is then injected through the cement towers bilaterally under flouro to ensure no cement leakage. Wounds are irrigated, sutured and dressed. Patients perform weight-bearing activities as privileges if tolerated on postoperative day 1.

## 4. Results

A total of six patients with sacral insufficiency fractures who failed conservative management were initially identified for inclusion. Following a comprehensive chart review, one patient was excluded due to an incomplete medical record. Consequently, the final analysis cohort comprised five patients.

The demographic profile of the cohort exhibited a 100% female distribution with a mean age of 78 years (range: 65–95 years). Anatomical and diagnostic assessment via computed tomography (CT) revealed that the majority of the cohort suffered from bilateral pathologies: four patients presented with bilateral sacral insufficiency fractures, and one patient presented with a unilateral fracture pattern.

Surgical intervention was tailored to the fracture laterality confirmed on preoperative imaging. Accordingly, four patients underwent bilateral robotic-assisted sacroiliac screw fixation and cement augmentation, and one patient underwent unilateral fixation, resulting in a total of nine fenestrated sacroiliac screws placed across the cohort. Cement augmentation was successfully completed in all nine screws without any instances of intraoperative cement abort protocols.

The mean operative time for the procedures was 36 min, with a total range spanning 15 to 47 min. Intraoperative radiation exposure, quantified by the number of fluoroscopy shots required for trajectory verification and cement delivery visualization, demonstrated a median of 13 shots per procedure (range: 6–19 shots). Detailed individual surgical and baseline metrics are structured in Table 1.

Postoperatively, the clinical protocol for immediate mobilization was successfully executed across the entire cohort. On postoperative day 1 (POD 1), 100% of the patients achieved independent or assisted ambulation under the direction of the institutional physiotherapy team. All patients reported a subjective, immediate reduction in mechanical and insufficiency-related pain during early mobilization compared to their preoperative baseline status.

All patients completed 2-week, 6-week and 3-month clinical and radiological follow-up. Radiographic parameters confirmed the strict maintenance of pelvic alignment and complete hardware integrity across all patients, with no signs of secondary displacement, as well as screw migration or loosening evident on multi-planar follow-up imaging.

In strict accordance with the primary safety endpoints defined in our methodology, there were no reported intraoperative or postoperative complications. Specifically, there were 0 cases of surgical wound infection, 0 cases of wound dehiscence requiring secondary surgical intervention, 0 cases of hardware failure, and 0 cases of fracture non-union. Furthermore, no clinical signs of neurovascular deficit or symptomatic cement extravasation in the neural foramina or pelvic cavity were observed in any of the five patients.

## 5. Case Example

A 76-year-old, independent and ambulatory female presented to the emergency department a week after a low-energy fall. She had a medical history of Parkinson’s and Type 1 diabetes, as well as breast cancer treated with biological therapy. A recent PET scan found no metastasis of her disease.

Following the fall, she continued walking for several days, but then gradually began developing lower back pain, which became debilitating to the point that she was non-ambulatory.

**Figure 1:**
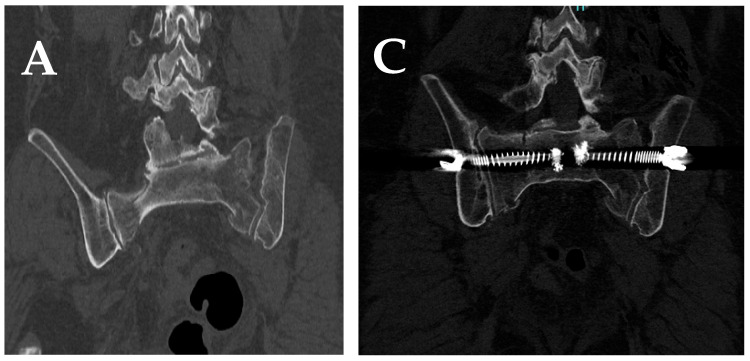

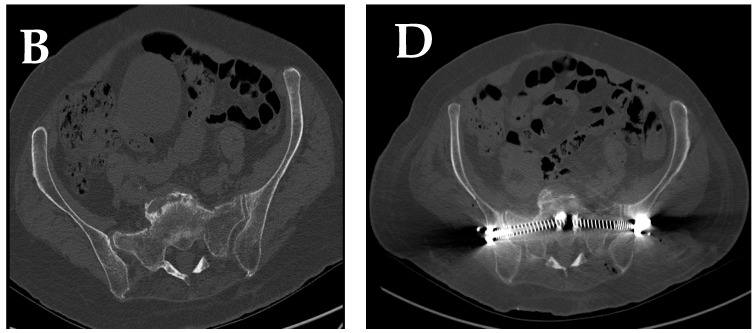
Preoperative (**A**,**B**) and postoperative (**C**,**D**) coronal and axial CT scan cuts.

**Figure 2:**
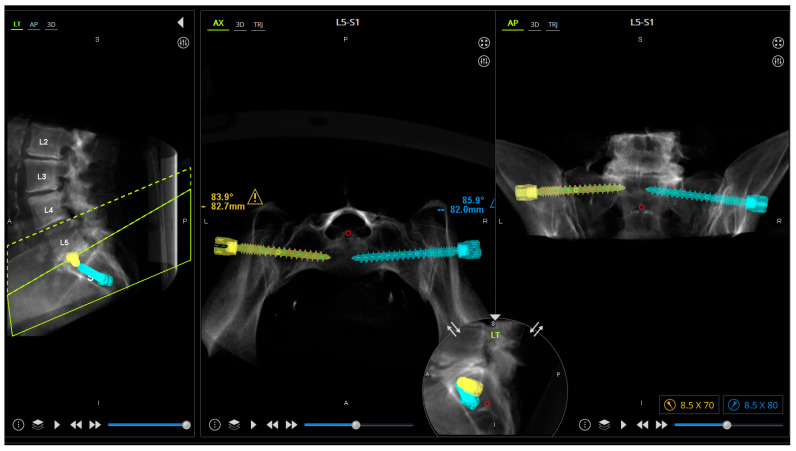
Screw planning using the Mazor stealth preoperative planning software.

On admission, she was diagnosed with a bilateral sacral insufficiency fracture and was initially treated conservatively with pain medication and physical therapy without significant improvement in her symptoms. She subsequently underwent robotic-assisted bilateral iliosacral fixation and cement augmentation. The patient’s pre- and postoperative imaging studies are portrayed in Figure 1. Surgical planning images are portrayed in Figure 2. Following surgery, the patient reported complete resolution of her pain and began ambulating on postoperative day 1.

## 6. Discussion

The increasing prevalence of sacral insufficiency fractures has brought this challenging clinical entity into the spine surgeons’ everyday practice. When untreated, this pathology can result in prolonged immobilization, secondary to severe pain, and subsequent medical complications and functional decline [13]. Current treatment strategies balance non-operative care against surgical intervention. While the former may suffice in a subset of patients, slower recovery and secondary complications have led many clinicians to opt for early surgical intervention [6,7,13]. There are two surgical methods typically applied for the treatment of sacral insufficiency fractures. Cement augmentation has been shown to provide immediate pain relief in certain cases [14,15]. This has been described as due to the immediate reduction in fracture micromotion [16]. For more complex injuries, including bilateral injuries and those with spinopelvic dissociation, fixation using percutaneous iliosacral screws is typically opted to achieve immediate stability and allow patient mobilization [7,17]. Pulley et al. [18] studied 16 patients with U-type sacral insufficiency fractures who were unable to mobilize and were treated with iliosacral fixation. They found an immediate decrease in the postoperative visual analog score of 3.2 points and mean postoperative day 1 walking distance of 114 and 88 feet in the acute and chronic surgery groups. However, conventional fluoroscopic-guided fixation techniques are technically demanding due to narrow anatomical corridors and adjacent neurovascular structures, with a well-documented risk of hardware malposition even in experienced hands [8,9,10].

The use of robotic navigation allows for pre-planning of screw trajectory and size, ensuring safe, precise screw placement while taking into account the unique iliosacral anatomy of each patient. This is especially critical when attempting optimal fixation in patients with poor bone stock or those with atypical fracture patterns or dysmorphic anatomy. The technical metrics presented in this study, including the proper placement of instrumentation and lack of neurological or vascular complications, demonstrate the utility of this method. Furthermore, the use of robotic navigation allows for a reduction in surgeon and patient radiation exposure, which is notoriously associated with traditional fluoroscopic techniques [19]. This is especially the case when using complete navigation from drill and tap to screw placement, as is the case with our surgical technique. An additional value of this technique lies in the possible synergistic effect of cement augmentation through the fenestrated screw. Thus, in addition to improving fixation stability and maximizing the local reinforcement of the osteoporotic bone, the patient can benefit from the analgesic effects of sacroplasty.

We believe that this surgical technique offers an efficient, minimally invasive and safe solution to a pathology that is potentially devastating to a high-risk and frail population. It allows for immediate postoperative pain relief and mobilization, thus countering the risks associated with immobility, as was demonstrated in our cohort. However, this is an initial series, and the interpretation of our findings should be viewed within the constraints and limitations of a case series. Furthermore, our study lacks specific pain measures, which could provide for a more meaningful analysis of results. While our findings demonstrate acute safety and efficacy, larger, multicenter randomized controlled trials are essential to validate these outcomes against traditional freehand techniques and conservative management over extended follow-up periods. Looking forward, the results observed in this initial cohort highlight several critical avenues for future research and broader clinical applications. Beyond sacral insufficiency fractures, the principles of this hybrid robotic and cement-augmented approach hold significant potential for expansion into other complex pelvic pathologies. Possible applications include the stabilization of pathologic fractures secondary to metastatic disease, where bone stock is similarly compromised. As robotic software and navigation platforms continue to evolve, integrating artificial intelligence for automated trajectory planning and predictive modeling of cement distribution could further refine this technique, ultimately standardizing complex pelvic surgeries and broadening access to minimally invasive solutions for highly vulnerable patient populations. However, a possible practical limitation is the shortcoming of this study in analyzing the financial aspect of this technique. As technology and innovation drive our surgical techniques and abilities forward, a cost–benefit analysis is warranted to assess the economic impact of technology adoption.

## Figures and Tables

**Table 1:** Patient demographics and surgical data.

Pt. #	Age	Sex	Fracture Pattern	Fixation	Time of Surgery	Flouro Shots	Complications
1	77	f	Bilateral insufficiency	Bilateral	36 min	7	None
2	95	f	Bilateral insufficiency	Bilateral	21 min	15	None
3	65	f	Bilateral insufficiency	Bilateral	41 min	6	None
4	76	f	Bilateral insufficiency	Bilateral	47 min	19	None
5	76	f	Unilateral insufficiency	Unilateral	15 min	13	None

## Data Availability

The original contributions presented in this study are included in the article. Further inquiries can be directed to the corresponding author(s).
